# Survival benefit for patients receiving antibiotics following out-of-hospital cardiac arrest

**DOI:** 10.1186/cc10883

**Published:** 2012-03-20

**Authors:** KJ Davies, ID Kerslake, J Walters, MJ Thomas

**Affiliations:** 1 Bristol Royal Infi rmary, Bristol, UK

## Introduction

Therapeutic hypothermia (TH) has become standard management following out-of-hospital cardiac arrest (OHCA). Recent evidence suggests TH increases the risk of pneumonia. We retrospectively assessed infective indicators after OHCA and evaluated the effect of antibiotics on survival.

## Methods

We identified all patients admitted to the ICU of a regional primary angioplasty hospital following OHCA from May 2007 to December 2010. We recorded ICNARC predicted mortality scores, blood and respiratory (protected catheter aspiration) culture results, white blood cell count (WBC) and C-reactive protein (CRP), hospital outcome and ICU length of stay. All chest radiographs (CXRs) were reviewed by a respiratory consultant (JW). Any antibacterial therapy was recorded.

## Results

A total of 144 patients were admitted to the ICU following OHCA. Mean age was 61.7 years (95% CI 59.0 to 64.4). The mortality rate was 66.67% (58.62 to 73.84) with mean ICNARC predicted mortality of 77.11% (73.84 to 80.39). Of 144 patients, 138 (95.8%; 91.1 to 98.1) had at least one positive marker of infection within 72 hours. Sixty-four had microbiology samples analysed, 34 of which were positive (53.1%; 41.1 to 64.8%). Of 88 patients who had a CXR, 26 (29.6%; 21.0 to 39.8) had consolidation. Ninety-six of 115 patients (83.5%; 75.6 to 89.1) had a CRP >100 mg/l (normal value <10 mg/l) within 72 hours and 82 of 115 (71.3%; 62.5 to 78.8) had an abnormal WBC (<4.0 or >11.0 × 10^9^/l). Fifty-six of 144 patients (38.9%; 31.3 to 47.0) received antibiotics during the first 7 days of their ICU stay (mean time to first dose 2.17 days; 1.69 to 2.66). The hospital mortality rate for these patients 53.6% (40.7 to 66.0) was significantly less than those not receiving antibiotics 75.0% (65.0 to 82.9) (χ^2 ^6.14, *P *= 0.01) with absolute risk reduction of 0.214 (0.055 to 0.365) and NNT of 5 (3 to 18). There was no difference in age (59.9 ± 4.2 vs. 62.9 ± 3.5) or ICNARC predicted mortality (75.1 ± 5.2 vs. 78.4 ± 4.2) between the groups.

## Conclusion

The post-arrest management of OHCA is commonly complicated by infections, the diagnosis of which is delayed by a universal increase in inflammatory markers, body temperature control, delay in the processing of samples and poor quality radiography. We have shown a significant reduction in mortality in patients receiving antibiotics compared with patients who do not, despite there being no difference in age or predicted mortality between the groups. This could be due to treatment of an aspiration pneumonia, an anti-inflammatory effect or that some patients did not survive long enough to receive antibiotics. It suggests that a formal clinical trial is warranted.

